# Validation of the Finnish Health Improvement Profile (HIP) with patients with severe mental illness

**DOI:** 10.1186/s12888-020-02511-5

**Published:** 2020-03-11

**Authors:** Camilla Werkkala, Maritta Välimäki, Minna Anttila, Virve Pekurinen, Daniel Bressington

**Affiliations:** 1 Department of Nursing Science, University of Turku, Joukahaisenkatu 3-5, 20014 Turku, Finland; 2 The Hong Kong Polytechnic University, Hung Hom, Hong Kong

**Keywords:** Severe mental illness, Physical health, Assessment, Validation

## Abstract

**Background:**

Physical health among people with severe mental illness (SMI) is a global concern. However, many people with SMI do not receive regular comprehensive health checks. There is currently no validated physical health check instrument systematically used in Finnish mental health services. Therefore, this study aims to validate and establish the potential clinical utility of the translated Health Improvement Profile (HIP) tool for Finnish patients with SMI and compare differences in physical health risk items across genders.

**Methods:**

The content validity of the two-way translated Finnish HIP (HIP-F) was evaluated by five nurses and four patients with SMI using cognitive debriefing (to assess the clarity and relevance of each item and the recommended actions of the HIP tool). The potential clinical utility was assessed using a pilot test involving 47 patients. The prevalence of red-flagged (risk) items in the whole sample, across female and male participants, and the frequencies of any type of missing item response were calculated and analysed using descriptive statistics. A chi-square test was used to determine differences in frequencies of red-flagged items across genders.

**Results:**

Based on the cognitive debriefing, the HIP-F was found to have moderate content validity regarding the clarity and relevance of the items and recommended actions (the average scale level content validity index, S-CVI/Ave, 0.74). In the pilot test, some missing item responses were identified, but in the sample, nurses identified 399 areas of health and health behaviour risks (mean 8.6 per patient) using the HIP-F. The most frequently red-flagged items were body mass index (BMI) and waist circumference (83.0%), smoking status (48.9%) and lipid levels (46.8%). Female patients had a higher frequency of red-flagged items than males in BMI (92.6% vs. 70.0%, *p* = 0.04) and waist circumference (96.3% vs. 65.0%, *p* = 0.01).

**Conclusions:**

The results demonstrate that the Finnish HIP has moderate content validity and preliminary clinical utility for evaluating the physical health and health behaviours of people with SMI. The HIP-F findings help to sign-post evidence-based interventions for identified areas of concern. Additional nurse training may be necessary to realise the potential clinical utility of the tool in Finland.

## Background

Severe mental illnesses (SMI) like schizophrenia, bipolar disorder and severe depression present many treatment-related challenges [1]. Patients treated with antipsychotics may suffer from obesity, which can lead to coronary artery disease, hypertension, type II diabetes mellitus, osteoarthritis and stroke [2–4]. Low physical activity, unhealthy diets, and smoking can further increase the risk for comorbidity of cardiovascular diseases, metabolic illnesses and cancer [5, 6]. Due to cardiovascular diseases, the mortality rates of people with schizophrenia are threefold compared to the general population [7].

A number of guidelines have emphasised the importance of monitoring physical health [8–10], and different instruments are used to assess physical health status among people with SMI. These instruments have focused on patient satisfaction level (SAS-SMI) [11], quality of life (i.e. EQ-5D) [12], WHOQOL-Brief [13], 15D [14], SF-12 [15]) or functioning (GAF) [16]. More recently, due to a recognised need for a more comprehensive approach, a physical health check tool (PHC) [17] has been developed for research purposes. However, the PHC does not provide clear recommendations for interventions or lifestyle advice, and therefore, its clinical utility may be limited [17].

The Health Improvement Profile (HIP) is a comprehensive clinical tool used to identify physical health-related risks, assess lifestyle behaviours and direct appropriate interventions [18]. The HIP findings enable staff working in mental health settings to profile each patient’s physical health state and direct possible health promotion interventions based on patients’ individual health needs [18]. The HIP was originally developed in the UK and has been translated and validated in Thailand [19], and Hong Kong [20, 21] demonstrating patient acceptability and clinical utility in these different international settings to identify areas where physical health interventions and lifestyle modifications are required [19, 21]. Although these studies did not report a formal validity test process or results, the study findings suggest that the HIP may be useful as a tool for engaging service users in conversations about their physical health, and for identifying areas of concern. For example, in Scotland, the use of the HIP identified a total of 189 health risk issues in a sample of 31 patients with SMI [22] and 352 issues in 105 participants in Thailand [19]. However, although White et al. (2010) supported the use of the HIP in UK clinical settings in earlier studies, a subsequent UK RCT study shows that it may not be feasible for the HIP to be fully completed by nursing staff in a research context [23]. These somewhat mixed findings may suggest that the utility and acceptability of the HIP is clinically and culturally context-specific, which highlights a need to conduct preliminary tests of utility and acceptability before the tool is used in a new treatment setting.

The tool uses a simple traffic light approach to highlight areas of identified risk (red, action required; green, healthy) in order to draw attention to areas of concern. According to the traffic light, individualised evidence-based recommendations and support can be offered to each patient to improve their physical health [24]. Given that the SMI patient population typically has low levels of engagement in physical health treatment [25], another benefit of the HIP tool is that it can support the patient’s participation in their own care because the HIP assessment process includes health monitoring in collaboration with a nurse [18, 20].

At present there is no validated physical health check instrument systematically used in Finnish mental health care services to guide patients and nurses toward physical health promotion interventions. This is an unmet need because patients with SMI have a high risk of physical health problems and premature deaths [26], especially in Finland, where patients with schizophrenia have a 2.5 to 3.5-fold higher risk of mortality compared to the general population due to somatic diseases [27, 28]. Risk factors that can lead people with SMI to experience physical health problems and premature death are numerous [26]. Outpatients with schizophrenia in Finland may have health care needs such as problems with blood glucose, dermatological, dental, cardiovascular, ophthalmological issues or gastrointestinal problems [29], including constipation and dyspepsia, which are associated with abnormal laboratory findings [30]. The most common major health issue is metabolic syndrome, which appears in more than half of patients with schizophrenia [31]. In Finland, alcohol-related mortality is common among patients with psychotic disorders. Although substance abuse is more common among men, alcohol-related mortality as a consequence of abuse is substantially higher among female patients with SMI [27]. There are also regional differences in mortality within the national schizophrenia population. Hospital districts’ resources, outpatient services, and treatment vary between regions, which may explain the regional variations [32]. Although these health problems in this population are well known in Finland, the monitoring and treatment of somatic issues are currently insufficient [29]. Annual health checks are recommended, but a systematic health examination protocol is missing, and nurses therefore may decide themselves which health issues they discuss with their patients. Therefore, a valid and easy-to-use tool is urgently needed to assess and promote individual patients’ physical health in mental health services. In this paper, we describe the translation process, and assess the content validity and potential clinical utility through a pilot test of the Finnish version of the HIP tool (HIP-F). To the best of our knowledge, this is the first time a structured comprehensive physical health assessment tool such as the HIP has been validated and used with the SMI population in Finland.

## Study AIMS

This study’s aims are two-fold: first, to translate and establish the content validity of the HIP-F for Finnish patients with SMI; second, to assess the potential clinical utility of the tool by calculating the prevalence of identified red-flagged items in the whole sample, across female and male participants, and the frequencies of any type of missing item response.

## Methods

### Design

**Fig. 1:**
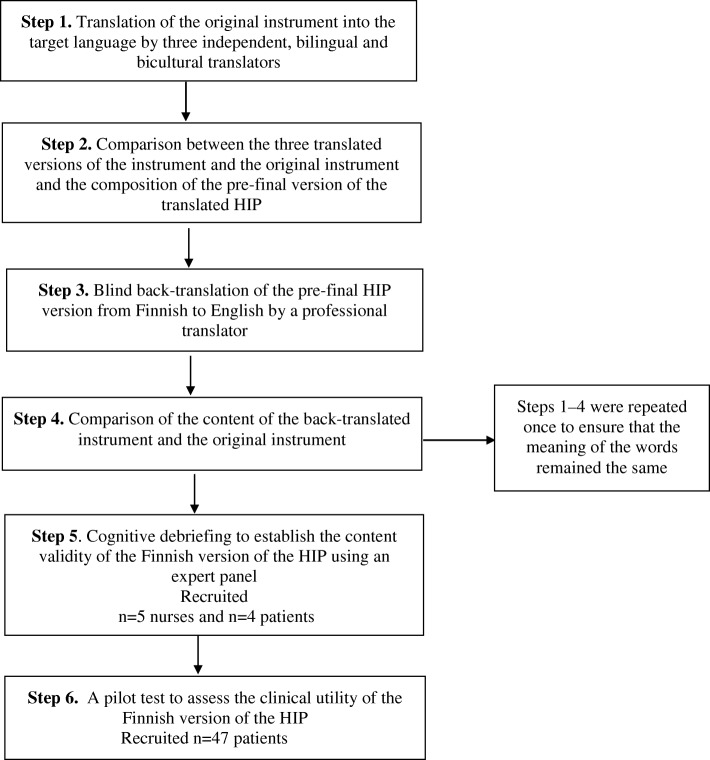
Summary of the study process

For the validation process, the study adapted the first six steps of a seven-step guideline developed by Sousa and Rojjanasrirat [33], for the translation, adaptation and cross-cultural validation of research instruments. The full process included 1) translation of the original instrument into the target language; 2) comparison of the three translated versions of the instrument; 3) a blind back-translation; 4) comparison of the back-translated version of the instrument with the original instrument; 5) cognitive debriefing (content validity assessment); and 6) a pilot test [33]. Step 7, a full assessment of psychometric properties (such as correlation coefficients, scale and item analysis and calculation of sensitivity) is not appropriate for the HIP as it is a comprehensive clinical health check tool. Therefore, we describe the translation process (Steps 1–4), the results of the cognitive debriefing (Step 5) and the pilot test (Step 6). A summary of the study process is described in Fig. 1.

### Setting

The study was carried out in three psychiatric out-patient clinics in southern Finland during December 2017 to April 2018. The cognitive debriefing was conducted in two outpatient clinics and the clinical utility pilot testing was conducted in another outpatient clinic that also provided intensive home care. These clinics were selected because they provided both crisis and long-term, recovery-focused mental health care services by multidisciplinary teams (psychiatrists, social workers, mental health nurses) for 460,000 inhabitants in the catchment area [34]. The patients’ frequency of attendance at the clinic depended on their agreed treatment plan. In the outpatient clinics, all patients had been diagnosed with a range of psychotic disorders (F20–29) [35]. The nature of the nurses’ work in mental health services and patients’ frequent visits to the clinics were considered to offer an ideal opportunity for monitoring patients’ physical health and supporting their healthy lifestyle for the instrument validation [18, 22, 36]. Moreover, since cognitive problems may be associated with SMI, we aimed to ensure that each nurse recruited patients with cognitive orientation and the capacity to give informed consent for their study participation [37, 38].

### Description of the HIP

The HIP tool has 27/28 items (28 items for the female version). The items include a range of physical health parameters (e.g., body mass index, lipid levels) and health behaviours (e.g. diet, smoking status). The responses of each item are marked based on the discussion between the patient and staff, and data recorded in the patient’s medical records, and the results are categorised as red (action required) or green (healthy). Based on this traffic light system, the nurse can choose the recommended action to implement health promotion, which can range from encouraging lifestyle changes and conducting blood tests and electrocardiograms to referring the patient for further care. The HIP has been developed to be used in outpatient clinics at least annually among persons with SMI [18, 36]. The tool includes a manual, which offers more detailed information about the physical health parameters and recommended actions, cut off points, and guidance about the recommended actions [18].

### Translation process

First, with the permission of the authors, the original HIP tool was translated from the original English version into Finnish by three independent, bilingual and bicultural translators with distinct backgrounds in health care and research and whose native language was the desired target language of the instrument [33]. Second, the three independent translated versions of the instrument were compared with the original instrument by three independent translators regarding words, meanings and sentences (five rounds in total) and the pre-final version of the translated HIP in the target language was composed. Third, the blind back-translation of the pre-final HIP version from Finnish to English was conducted by a professional translator who was not knowledgeable about the health care terminology and was not familiar with the original instrument [33]. Fourth, the content of the back-translated and the original instrument was compared, focusing on similarity of the items and response format regarding wording, sentence structure, meaning and relevance [33]. A committee of the three health professionals involved in Steps 1 and 2, and one co-author experienced in translation processes [20, 21] collaborated with the original developer of the instrument. To ensure that the meaning of words remained the same, Steps 1–4 were repeated once [33]. Finally, the back-translated Finnish version of the HIP was subjected to cognitive debriefing to establish content validity in this study, and it was accepted by its copyright authors.

### Cognitive debriefing to establish content validity

Cognitive debriefing was performed to establish the content validity, i.e., the extent to which an instrument represents all facets of a given construct it is purposed to measure. This was done by assessing the clarity and relevance of each item and the recommended actions of the HIP tool in the target language. Any potential challenges concerning the measure were also identified [39]. An expert panel consisted of 6–10 experts. The experts were nurses who should be knowledgeable about the content areas of the instrument (possible users of the instrument) and patients (possible target population and users of the instrument together with nurses). The expert panel assessed the conceptual and content equivalence (content-related validity) using Content Validity Indexes (I-CVI, S-CVI) [33]. Participants who rated any item of the instrument as unclear or irrelevant were asked to provide suggestions as to how to rewrite the item.

#### Recruitment and participants

For nurses, the total population sampling method was utilised [40, 41]. All nurses (*N* = 15) who worked in the selected two study units and who fulfilled the following criteria were recruited into the study: professional education (a nurse, mental health nurse), permanently employed or a long-term temporary worker, currently working in clinical practice as a patient primary nurse in coordinating and providing care for patients [42].

Patients are recommended to be members of expert panels because they bring added value with their experience as a service user of mental health care services [43]. Purposive sampling was used to recruit the patients in the expert panel. Each nurse (*N* = 15) on the ward was asked to recruit one patient (*N* = 15) who was visiting the out-patient clinic regularly (totally 15 patients). Patients were recruited if they were minimum 18 years old, able to read and speak Finnish, and able and willing to give written informed consent. All recruited patients were diagnosed with a psychotic disorder (F20–29) [35].

#### Instrument and the data collection

To determine the validity of the items and recommended actions, a specific instrument was utilised for assessment of the clarity and relevance of the HIP items and recommended actions. The instrument used a 4-point Likert scale (1 = not clear/relevant, 2 = moderate clear/relevant, 3 = very clear/relevant, 4 = extremely clear/relevant) [33, 44]. There was also a possibility to provide suggestions for how to modify items in cases of any unclear or irrelevant content. For nurse respondents, a link with access to the electronic survey was sent in an email message. For patients, the data collection was conducted with paper format questionnaires. The patients answered the questionnaire independently after scheduled meetings with the nurses.

#### Data analysis

The Content Validity Index (CVI) was assessed to establish the degree of the clarity and relevance of the items and recommended actions of the HIP [45]. Three approaches were used to analyse the content validity of the tool. First, the item content validity index (I-CVI) regarding the clarity and relevance of the items and recommended actions were calculated (the value varied between 0 and 1.0). Congruence was computed as the number of experts giving a rating of “3 = very clear/relevant” and “4 = extremely clear/relevant” for each item divided by the total number of experts. Likewise, the scale level content validity index (S-CVI) was calculated using the number of items in the HIP that achieved a rating of “3 = very clear/relevant” and “4 = extremely clear/relevant” [44, 45]. Hence, a 4-point Likert scale turned into a trichotomous scale, where ratings of 1 and 2 were considered content invalid while ratings of 3 and 4 were considered content valid [44, 45]. Second, the average value of the I-CVI (S-CVI/Ave) was calculated by totalling all the I-CVI values of each item and dividing this by the number of items. Third, the total S-CVI of the HIP was analysed by calculating the S-CVI/Ave values and distributing the result among four average value (S-CVI/Ave) of the I-CVI values (the value commonly accepted based on 6 or more expert panel members is 0.78) [44, 45].

### Pilot test to assess the potential clinical utility

Based on the recommendations by Sousa and Rojjanasrirat [33], the final version of a translation should be tested regarding its psychometric properties with pilot testing among a bilingual sample from the target population in which the instrument will be used. This is to establish criterion equivalency and further support the semantic, conceptual, construct and content equivalency of the pre-final version [33]. However, since the HIP tool is a profiling tool, not all statistical methods adopted in the evaluation of psychometric properties and reliability of instruments were appropriate in our study. Therefore, the potential clinical utility of the tool was assessed in this study using a pilot test for patients with SMI with a final version of the HIP in Finnish. This was assessed as follows: 1) the prevalence of red-flagged items identified in the total sample was calculated, 2) red-flagged items across female and male participants were compared, 3) and frequencies of missing item responses (single missing item responses, all questionnaires with partly missing item responses) were calculated.

#### Recruitment and participants

The study population for the pilot testing consisted of patients with SMI who had regular visits with a nurse at one of the out-patient clinics. Convenience sampling was used to recruit patients who were eligible to participate in the study [40, 41]. Each of the 17 nurses at the clinic were asked to recruit at least 5 patients with SMI (*N* = 85) who were at least 18 years old, able to read and speak Finnish, and able and willing to give written informed consent. The target sample size was 30–40 participants for accomplishing the statistical analysis [46]. We invited 85 patients to participate and finally conducted the pilot testing with a sample of 47 patients (a response rate of 55%).

#### Data collection

Nurses evaluated the physical health state and health behaviours of patients who had a scheduled visit in the outpatient clinic during the data collection period (February–April 2018). Nurses and patients filled in a paper version of the HIP-F tool. Staff extracted patients’ cholesterol and glucose values, blood pressure and pulse from medical records to avoid any duplication of effort in data collection [20]. The following demographic and clinical characteristics were recorded during the visit: age, gender, height, weight, waist circumference and temperature. To assist in this task, nurses used the Finnish version of the translated HIP manual for this study.

#### Data analysis

Descriptive statistics mean, median (Md), interquartile range (IQR) and standard deviation (SD) were used to describe the participant characteristics and to identify the prevalence of red-flagged items using the cut off points of the HIP tool [19–21]. A chi-square test was conducted to determine differences in frequencies of red-flagged items across genders. An independent samples t-test for normally distributed data and a non-parametric Mann-Whitney U-test for non-normally distributed data were used to measure significant differences in physical health issues across genders [41]. A two-sided level of significance was set at *p* ≤ 0.05 for all tests. The clinical utility of the HIP regarding missing items was analysed using descriptive statistics (frequencies, valid percentages) of single missing item responses and partially missing item responses [42, 47]. IBM SPSS version 24 for Windows was used for all analyses.

## Results

### Characteristics of the participants

For the cognitive debriefing (content validity) assessment, out of 15 possible nurses and 15 patients, nine persons agreed to participate in the cognitive debriefing panel (5 nurses, 4 patients), a response rate of 33 and 27% respectively. All nurse participants were women. Their mean age was 45.20 (SD 13.91) years. The average length in experience of working in mental health care was 21.20 years (SD 12.71). In the patient sample, half were women (*n* = 2) and half were men (*n* = 2). Their mean age was 41.30 (SD 8.05) years. Based on patients’ medical records, all patient participants had a diagnosis categorised with ICD-10 (F20–29).

For the assessment of clinical utility, out of 47 patients, 27 were female (57.4%) and 20 were male (42.6%). Their age range was 23–69 years, and the mean age was 41.10 years (SD 12.17). All participants had a diagnosis categorised with ICD-10 (F20–29).

### The cognitive debriefing

#### Clarity of the items and recommended actions

Nurses and patients evaluated the clarity of each HIP item and recommended actions. Items that were considered very clear (I-CVI 0.89) were waist circumference, pulse, liver function tests and bowels. Items considered moderately clear (I-CVI 0.78) were temperature, lipids levels, glucose, breast self-examination, menstrual cycle and smoking status. The least clear items (I-CVI 0.44) were prostate and testicles, fluid intake, caffeine intake, and sexual satisfaction (Table 1).

**Table 1:** Content Validity Indexes

Item	Clarity I-CVI	Recommended action Clarity I-CVI	Relevance I-CVI	Recommended action Relevance I-CVI
BMI	0.56	0.78	0.67	0.78
Waist circumference	0.89	0.78	1.00	0.89
Pulse	0.89	1.00	1.00	0.78
Blood pressure	0.67	0.89	0.89	0.89
Temperature	0.78	0.56	0.78	0.67
Liver function tests (in last 3 months)	0.89	0.78	1.00	1.00
Lipid levels	0.78	0.67	0.89	0,67
Glucose	0.78	0.78	1.00	1.00
Cervical smear (f)	0.67	0.67	1.00	0.89
Prostate and testicles (m)	0.44	0.44	0.67	0.67
Sleep	0.67	0.78	0.67	0.89
Teeth	0.67	0.78	0.78	0.78
Eyes	0.67	0.89	0.78	0.89
Feet	0.67	0.89	0.56	0.89
Breast self-examination (female and male)	0.78	0.67 (f) 0.33 (m)	1.00	1.00 (f) 0.89 (m)
Menstrual cycle (f)	0.78	0.44	0.89	0.78
Smoking status	0.78	1.00	0.78	1.00
Exercise	0.67	0.89	0.78	0.89
Alcohol intake	0.67	0.89	0.56	0.89
Diet: 5 portions a day	0.67	0.89	0.56	0.89
Diet: fat intake	0.56	0.56 (f) 0.56 (m)	0.78	0.78 (f) 0.56 (m)
Fluid intake	0.44	0.78	0.67	0.89
Caffeine intake	0.44	0.56	0.34	0.67
Cannabis use	0.67	0.56	0.56	0.67
Safe sex	0.56	0.67	0.56	0.67
Urine	0.56	0.56	0.45	0.78
Bowels	0.89	0.78	0.89	0.89
Sexual satisfaction	0.44	0.44 (f) 0.56 (m)	0.67	0.67 (f) 0.67 (m)
**S-CVI/Ave**	**0.68**	**0.70**	**0.76**	**0.82**

*BMI* body mass index, *I-CVI* item content validity index, *f* female, *m* male

The recommended actions considered very clear (I-CVI 0.89) were blood pressure, eyes, feet, exercise, alcohol intake, and diet: 5 portions a day. For example, regarding eyes, it was advised to refer the patient for an eye check (“Prompt to self-refer/refer to optometrist if no eye check”) if it had not been conducted in the last 2 years. Items for actions relating to BMI, waist circumference, liver function tests, glucose, sleep, teeth, fluid intake and bowels were evaluated as moderately clear (I-CVI 0.78). The least clear recommended actions (I-CVI 0.44) were prostate and testicles check (Table 1).

No recommendations were provided to rewrite the items to make them clearer.

#### Relevance of the items and recommended actions

Nurses and patients evaluated the relevance of each HIP item and recommended actions. Items considered extremely relevant (I-CVI 1.00) were waist circumference, pulse, liver function tests, glucose, cervical smear, and breast self-examination. Items considered very relevant (I-CVI 0.89) were blood pressure, lipid levels, menstrual cycle and bowels, while items evaluated as moderately relevant (I-CVI 0.78) were temperature, teeth, eyes, smoking status, exercise and diet: fat intake. The least relevant item (I-CVI 0.34, accordingly) was caffeine intake.

Recommended actions seen as extremely relevant (I-CVI 1.00) were related to blood pressure, liver function tests, glucose, breast self-examination for females and smoking status. For example, regarding blood pressure it was advised that if a patient was overweight, they should lose weight (“Advice on weight loss (if overweight) and increased activity, reduction in alcohol intake, improved diet and smoking cessation”). Items considered very relevant (I-CVI 0.89) were waist circumference, blood pressure, cervical smear, sleep, eyes, feet, breast self-examination for males, exercise, alcohol intake, diet: 5 portions a day, fluid intake and bowels. Moreover, BMI, pulse, teeth, menstrual cycle, diet: fat intake for female and urine were evaluated as moderately relevant (I-CVI 0.78). The least relevant (I-CVI 0.56) recommended action related to diet: fat intake for males (e.g. “advice on reducing fat intake and achieving a well-balanced diet”) (Table 1).

The S-CVI/Ave of the clarity of the items was 0.68, and the S-CVI/Ave of the clarity of the recommended actions was 0.70. The S-CVI/Ave of the relevance of items was 0.76, and the S-CVI/Ave of the relevance of recommended actions was 0.82. Finally, the average of these four S-CVI/Ave values was 0.74. No suggestions for how to modify items in cases of irrelevant content were provided.

### The pilot test

In pilot testing, the potential clinical utility of the tool was assessed by calculating the prevalence of identified red-flagged items in the whole sample, red-flagged items across female and male participants, and frequencies (number and valid percentages) of any type of missing item response.

#### Red-flagged items

Out of all 47 participants, a total of 399 physical health issues were identified. The mean number of red-flagged items per patient was 8.6 (SD 3.12). Every patient had at least one item that was flagged red (range 3–18). Out of all participants, 83.0% had items BMI and waist circumference red-flagged. About half (48.9%) of the patients received red flags for smoking and 46.8% for unhealthy lipid levels. For fewer than half of the participants (44.7% red-flagged), over 2 years had passed since their last eye check. Further, 40.4% had bowel issues, such as diarrhoea, constipation, excessive urgency and straining or laxative use. Liver function tests had not been appropriately carried out for 40.4% of participants, whereas 38.3% of the participants had sleep problems and 36.2% reported sexual dissatisfaction. About one-fifth of the participants had hypertension (21.3% red-flagged), while the pulse rate for about one-third was too high (29.8% red-flagged) or glucose levels were too high (29.8% red-flagged). Regular breast checks had not been conducted as recommended for 38.3% of the sample (Table 2).

**Table 2:** Cut off points of HIP, red-flagged, green-flagged and unanswered items

Item	Red-flagged % (n)	Green-flagged % (n)	Unanswered % (n)
**BMI** < 18.50 > 25.00	83.0 (39)	17.0 (8)	0 (0)
**Waist circumference** ≥ 80 cm (f)/≥ 94 cm (m)	83.0 (39)	17.0 (8)	0 (0)
**Smoking status** Passive smoker/smoker	48.9 (23)	51.1 (24)	0 (0)
**Lipid levels** TC ≥ 6.2 mmol/L LDL – C ≥ 4.1 mmol/L HDL – C < 1.3 mmol/L TG ≥ 2.2 mmol/L)	46.8 (22)	51.1 (24)	2.1 (1)
**Eyes** > 2 years since last check	44.7 (21)	53.2 (25)	2.1 (1)
**Bowels** Diarrhoea, constipation, excessive urgency, straining, laxative use	40.4 (19)	57.4 (27)	2.1 (1)
**Liver function tests** > 3 months	40.4 (19)	59.6 (28)	0 (0)
**Breast self-examination (male and female)** Never self-check	38.3 (18)	61.7 (29)	0 (0)
**Sleep** < 3 h /> 8 h	38.3 (18)	61.7 (29)	0 (0)
**Sexual satisfaction** Dissatisfied	36.2 (17)	59.6 (28)	4.3 (2)
**Diet: fat intake** ≥ 2 portions a day	34.8 (16)	63.8 (30)	2.1 (1)
**Glucose** ≥ 7.0 mmol/L	29.8 (14)	66.0 (31)	4.3 (2)
**Pulse** < 60 bpm/> 100 bpm	29.8 (14)	68.1 (32)	2.1 (1)
**Safe sex** Inconsistently/Never	27.7 (13)	72.3 (34)	0 (0)
**Exercise** None	27.7 (13)	72.3 (34)	0 (0)
**Teeth** ≥ 2 years since last check	25.5 (12)	74.5 (35)	0 (0)
**Diet: 5 portions a day** ≤ 2 portions a day	25.5 (12)	72.3 (33)	2.1 (1)
**Blood pressure** ≥ 140/90 mmHg	21.3 (10)	78.7 (37)	0 (0)
**Menstrual cycle**^**(1**^ Irregular/Absent/Reduced/Excessive	21.3 (10)	34.0 (16)	3.7 (1)
**Fluid intake** < 1 l/day/> 3 l/day	19.1 (9)	80.9 (38)	0 (0)
**Cervical smear (f)** > 3 years (age 25–64) /> 5 years (age 50–64)	14.9 (7)	40.4 (19)	3.7 (1)
**Prostate and testicles (m)** Never self-check	14.9 (7)	27.7 (13)	0 (0)
**Feet** Never self-check	14.9 (7)	83.0 (39)	2.1 (1)
**Caffeine intake** ≥ 600 mg/day	14.9 (7)	85.1 (40)	0 (0)
**Temperature** < 36 °C/> 37.5 °C	10.6 (5)	89.4 (42)	0 (0)
**Alcohol intake** > 3 units/day (f), > 4 units/day (m)	8.5 (4)	89.4 (42)	2.1 (1)
**Urine** < 1 l/day/> 2 l/day	8.5 (4)	91.5 (43)	0 (0)
**Cannabis use** Occasional/Regular	4.3 (2)	93.6 (44)	2.1 (1)

*f* female, *m* male, *BMI* body mass index, *TC* total cholesterol, *LDL* low-density cholesterol, *HDL* high-density cholesterol, *TG* triglycerides

#### Comparison of red-flagged items for female and male participants

A statistically significant difference between females and males was found in only two items. First, females had more red-flagged health concerns related to BMI (92.6% vs. 70.0%, *p* = 0.04). Second, females had a higher frequency of red-flagged waist circumference values (96.3% vs. 65.0%, *p* = 0.01) (Table 3). Regarding health parameter indexes, only one difference between gender was found: females had higher BMI measurements than males (33.5 vs. 29.1, *p* = 0.04) (Table 4).

**Table 3:** Red-flagged items across genders

Item	Female ^(1^% (n)	Male ^(1^% (n)	• (df) p^2^
BMI	92.6 (25)	70.0 (14)	*x*^2^ = 4.15 (df 1), *p* = 0.04*
Waist circumference	96.3 (26)	65.0 (13)	*x*^2^ = 7.97 (df 1), *p* = 0.01*
Smoking status	48.1 (13)	50.0 (10)	*x*^2^ = 0.02 (df 1), *p* = 0.90
Lipid levels	51.9 (14)	42.1 (8)	*x*^2^ = 0.43 (df 1), *p* = 0.52
Eyes	40.7 (11)	52.6 (10)	*x*^2^ = 0.64 (df 1), *p* = 0.43
Bowels	48.1 (13)	31.6 (6)	*x*^2^ = 1.26 (df 1), *p* = 0.26
Liver function tests	40.7 (11)	35.0 (7)	*x*^2^ = 1.45 (df 2), *p* = 0.48
Breast self-examination (male and female)	29.6 (8)	50.0 (10)	*x*^2^ = 2.02 (df 1), *p* = 0.16
Sleep	48.1 (13)	25.0 (5)	*x*^2^ = 2.60 (df 1), *p* = 0.11
Sexual satisfaction	36.0 (9)	40.0 (8)	*x*^2^ = 0.08 (df 1), *p* = 0.78
Diet: fat intake	30.8 (8)	40.0 (8)	*x*^2^ = 0.43 (df 1), *p* = 0.52
Glucose	33.3 (9)	27.8 (5)	*x*^2^ = 0.16 (df 1), *p* = 0.69
Pulse	22.2 (6)	42.1 (8)	*x*^2^ = 2.08 (df 1), *p* = 0.15
Safe sex	29.6 (8)	25.0 (5)	*x*^2^ = 0.12 (df 1), *p* = 0.73
Exercise	25.9 (7)	30.0 (6)	*x*^2^ = 0.10 (df 1), *p* = 0.76
Teeth	22.2 (6)	30.0 (6)	*x*^2^ = 0.37 (df 1), *p* = 0.55
Diet: 5 portions a day	23.1 (6)	30.0 (6)	*x*^2^ = 1.73 (df 2), *p* = 0.42
Blood pressure	22.2 (6)	20.0 (4)	*x*^2^ = 0.34 (df 1), *p* = 0.85
Fluid intake	18.5 (5)	20.0 (4)	*x*^2^ = 0.02 (df 1), *p* = 0.90
Feet	19.2 (5)	10.0 (2)	*x*^2^ = 0.75 (df 1), *p* = 0.39
Caffeine intake	14.8 (4)	15.0 (3)	*x*^2^ = 0.00 (df 1), *p* = 0.99
Temperature	11.1 (3)	10 (2)	*x*^2^ = 0.02 (df 1), *p* = 0.90
Alcohol intake	11.5 (3)	5.0 (1)	*x*^2^ = 0.61 (df 1), *p* = 0.44
Urine	7.4 (2)	10.0 (2)	*x*^2^ = 0.10 (df 1), *p* = 0.75
Cannabis use	0 (0)	10.0 (2)	*x*^2^ = 2.72 (df 1), *p* = 0.10

^1^Cross-tabulation

^2^Chi-square test and *p*-value

*Statistical significance at *p* < 0.05

BMI, body mass index

**Table 4:** Physical health parameters across genders

Item	Female Mean (SD) ^a^ Md (IQR) ^b^	Male Mean (SD) ^a^ Md (IQR) ^b^	*p*-value
BMI^a^	33.5 (7.02)	29.1 (6.40)	0.04*
Temperature^a^	36.4 (0.47)	36.4 (0.39)	0.93
LDL cholesterol^a^	2.8 (0.84)	3.1 (1.07)	0.61
Waist circumference^b^	110.0 (22)	100.1 (28)	0.15
Pulse^b^	78.0 (11)	79.0 (39)	0.47
Systolic blood pressure^b^	114.0 (15)	119.0 (11)	0.31
Diastolic blood pressure^b^	77.0 (14)	81.0 (21)	0.88
Total cholesterol^b^	4.7 (1.8)	5.2 (2.6)	0.82
HDL cholesterol^b^	1.4 (0.4)	1.2 (0.8)	0.07
Triglyserids^b^	1.5 (1.2)	1.9 (2.9)	0.46
Glucose^b^	5.9 (1.4)	6.2 (1.3)	0.50

^a^Independent samples t-test, mean (SD, standard deviation) and p-value

^b^Non parametric Mann-Whitney U-test, Md, median (IQR, interquartile range) and *p*-value

*Statistical significance at *p* < 0.05

*BMI* body mass index.

*LDL* low-density cholesterol.

*HDL* high-density cholesterol.

#### Missing item responses

Single missing responses in questionnaires were found in the items for feet, lipid levels, eyes, bowels, menstrual cycle, cervical smear, diet: fat intake, diet: 5 portions a day, breast, pulse, glucose, sexual satisfaction, alcohol intake, and cannabis use. In 57.4% (*n* = 27) of the questionnaires, the numerical value of the health parameter was missing, although the item was appropriately green or red-flagged.

## Discussion

In this study, we aimed to validate the HIP tool (HIP-F) for Finnish patients with severe mental illness (SMI), and to assess its potential clinical utility by calculating the prevalence of red-flagged items identified in the whole sample, comparing the number of red-flagged items across female and male participants, and ascertaining the frequencies of any type of missing item response.

We found that the items related to glucose, blood pressure and pulse were identified to be very clear and very relevant. The relevance of these items was also supported by the number of participants who had specific health risks in these areas. Participants in our study had higher pulse rates (29.8%) than participants in previous HIP studies in Thailand (16.0%) [19] and Hong Kong (1.4%) [22]. Similarly, elevated glucose values appeared more often (29.8%) in our study sample than in Hong Kong (10.8%) [21] and Scotland (9.7%) [22]. Prevalence of hypertension in our sample, i.e. 21.3%, was similar to HIP findings in Hong Kong (21.6%) [21], while in Thailand, hypertension appeared in only 3% of study participants [19]. This high prevalence of risk is concerning because people with abnormal glucose values and hypertension are at high risk for comorbidity to cardiovascular diseases, metabolic illnesses and cancer, which all result in excess mortality in patients with SMI [5, 6]. Moreover, type II diabetes is one of the most common non-communicable diseases in Finland, and the prevalence is estimated to rise to around half a million inhabitants by 2030 [48].

We found that the percentage of participants with an unhealthy BMI value (80.0%) was much higher in Finland than that which was reported in Thailand (44.0%) [19], but the value in Finland was somewhat similar to that in Hong Kong (76.4%) [21] and Scotland (77.4%) [22]. Females reported more unhealthy BMI values (92.6% vs. 70.0%, *p* = 0.04) and waist circumference measurements (96.3% vs. 65.0%, *p* = 0.01) compared to males. This is in line with a study conducted in Thailand by Thongsai et al. [19], in which 51.0% of females and 38.0% of males had unhealthy BMI values. Health statistics in Finland have already reported that overweight is a serious problem in the general population. For example, in 2018, 21% of men and 20% of women between the ages of 20 and 64 years were found to be obese [49]. Other reports have also shown that 23–58% of the adult population (> 20 years old) in Finland is overweight [50, 51]. However, in our study the item concerning BMI was not assessed as relevant or clear in the content validity testing despite rates of overweight or central obesity in patients with SMI seeming to be higher than in the general Finnish population. Similarly, previous studies have already shown that many people with SMI have a poor diet and adopt other unhealthy lifestyle choices [37, 38, 52]. Indeed, the least relevant recommended action for male in our data was related to men’s diet (‘fat intake’). The finding is concerning because in the pilot testing 40.0% of the men reported a diet that included at least two portions of fat per day, which is a high rate compared to the previous study in Thailand (27.0%) [19]. Out of the total sample, 46.8% had a red-flagged item for lipid levels, which is a common problem in patients with SMI [5, 29]. The numbers of participants with a red-flagged lipid level in the current study are also higher than in previous HIP studies conducted in Thailand [19] and Scotland [22], highlighting a clear need for health promotion action among Finnish patients with SMI.

The reasons for the relatively high rates of overweight, central obesity and hyperlipidaemia in the current study, compared to previous HIP studies, may partially relate to differences in eating habits because Finnish food typically includes more fat than e.g. traditional Asian diets [20]. The impact of a high-fat diet may also be exacerbated by antipsychotic induced weight gain and the fact that some genetic predisposition for obesity has been reported in the Finnish population [53, 54]. Furthermore, studies have found associations between excess bodyweight and bowel issues such as constipation, which is a common side effect of some antipsychotic medications that can be compounded by an unhealthy diet [55]. In our study, the item concerning bowel functioning was evaluated as extremely clear and relevant, and in the pilot test, 40.4% of participants had the item red-flagged, which is higher than in previous HIP studies conducted in Thailand (15.0%) [19], Scotland (13%) [22] and in Hong Kong (15.9%) [56].

The item relating to prostate and testicles checks was rated as having low clarity and moderate relevance in the HIP-F content validity testing. The result is somewhat surprising because prostate cancer is the most common cancer among males in Finland [57], although the risk for prostate cancer may be lower among patients with schizophrenia compared to the general population [58]. The doubts about the clarity of this item may relate to differences in how prostate cancer is screened in Finland and in the UK (where the original HIP was devised). In the UK, using the PSA blood test as part of a screening programme has been discussed, and large international trials are being conducted to assess whether prostate screening could be helpful in identifying people at risk of prostate cancer [59]. However, according to the Finnish Current Care Guidelines, regular prostate screening is not required, and it is recommend for men only if they have had one or more prostate cancer occurrence in their family or if prostate cancer has been diagnosed in a sibling under 55 years old [60].

The item on sexual satisfaction was also evaluated to be one of the most unclear and irrelevant items, even though sexual health has been reported as a major concern for people with SMI [61], and sexual health and satisfaction are important elements of quality of life [62]. Despite the low perceived relevance of the sexual health items, over a third (36%) of participants reported sexual dissatisfaction and over a quarter (27.7%) had red-flagged items related to safe sex, which is more than in Thailand (14.0%) [19], Scotland (6.5%) [22] or Hong Kong (8.7%) [56].This finding seems to concur with previous studies reporting that people with SMI are more likely to engage in high-risk sexual behaviours than the general population [63, 64]. Given the apparent sexual health needs of many study participants, Finnish mental health nurses should be encouraged to provide sexual health advice and support clients in improving the quality of their intimate sexual relationships [65]. Unfortunately, sexual health does not seem to be addressed in usual routine clinical practice in many mental health services, and it has been reported that some nurses feel that this topic is intrusive and potentially damaging to the therapeutic alliance [61]. Furthermore, Finland may not be the only country struggling with these challenges. For example, in Thailand, the item related to sexual satisfaction was completely left out of the Thai HIP version because it was considered to be an embarrassing topic that might jeopardise the trust in the relationship between the nurse and the patient [19]. This logic is particularly dubious because nurses should be confident and skilled in discussing a range of sensitive issues with clients, such as self-harm, suicide and experiences of sexual abuse [61, 63].

Substance use was found to be relatively uncommon in the current study; only two men (10.0%) reported cannabis use. Moreover, only 4.0% of the participants, one male (5.0%) and three females (11.5%), reported drinking unhealthy levels of alcohol, which is a far lower percentage than has been found in HIP studies in Scotland (19%) [22], Thailand (14.0%) and Hong Kong (2.9%) [56]. Our findings seem to contradict earlier studies suggesting that people with SMI have high rates of comorbid alcohol and substance use [63]. However, it is possible that study participants may have underreported their use of alcohol. Although the average consumption of alcohol in Finland has decreased during the last years, in 2012 it was still one of the highest in European, with an annual average of 9.6 l per inhabitant [66]. On the contrary, the item related to smoking was evaluated as very clear and relevant, and it was red-flagged in 48.9% of the participants. Results seem to show that smoking may be more common among people with SMI in Finland than the general Finnish population (13%) [67], and also more prevalent than among people with SMI in Scotland 28% [22], Thailand 12% [19] and Hong Kong 27% [20]. Patients with SMI have previously been less likely to have been offered smoking cessation in primary care [68]. However, smoking cessation medication and other non-pharmacological support increases abstinence rates among those with mental health problems to as high as those in the general population and should therefore be offered to patients with SMI as a part of routine health promotion [69].

The item relating to eyes was evaluated as a moderately clear and relevant, but for 44.7% of participants over 2 years had passed since their last eye check. Eye checks were conducted less often in our sample than in previous HIP studies in Hong Kong (16.2%) [56], Scotland (12.9%) [22] and Thailand (10.0%) [19]. It is of great importance that people with SMI have regular eye checks because antipsychotic medication increases the risk for cornea and lens damage and has also been associated with cataract development [70]. Since patients with schizophrenia have remarkably weaker vision and participate vision checks essentially less frequently than the general population, they should be encouraged to routinely visit a local optician/optometrist, and regular eye evaluations should be included in physical health monitoring [70].

Our findings indicate that health screening for outpatients with SMI revealed a number of physical healthcare needs. If patients with SMI have difficulties in comprehending the impact of an unhealthy diet on their health and well-being, the health service system does not support preventive interventions, and if professionals do not value understanding patient health behavior and its impact of his or her well-being, patients’ health state may deteriorate [29]. For example, considering the prevalence of obesity in the adult population in Finland [50, 51] and physical health problems in patients with SMI, interventions should be urgently focused on those patients, especially those who are using antipsychotic medication. Health screening should contain physical examinations, analysis of previous medical history, laboratory tests (BMI, blood pressure) and assessment of lifestyle habits (dietary issues). A concern is that based on our study results nurses sometimes detected a health risk but they considered it to be unimportant. A screening tool has no value if nurses do not respond to an individual patient’s health needs. Therefore, nurses should be able to both identify patients’ health needs and select the most appropriate evidence-based intervention to improve patients’ physical health. This may require nurses to update their knowledge in order to guide patients towards adopting a healthier lifestyle. Supporting physically active lifestyles and helping patients to overcome psychological barriers to change their health behaviors could improve physical health outcomes and even facilitate functional recovery in SMI [25, 31].

In the pilot test there were partly answered single responses in 14 items out of 27/28 (~ 50%) and in over half of the questionnaires (57.4%). This may indicate that some nurses had problems filling out the tool as the item was rated appropriately with either a green or red flag, but the numerical level of the item was missing. This possibly reflects the importance of training for the nurses, since previous research results shows that many nurses do not have sufficient knowledge to evaluate and manage psychiatric patients’ physical health or that the evaluation is not considered to belong in mental health care [71]. However, our pilot study seems to show that the HIP-F is a tool with generally moderate clinical utility [see also 19,22] as it identified 399 health issues among 47 patients with SMI in Finland, compared to 189 health issues in a sample of 31 patients in Scotland [22] and 352 health issues in 105 participants in Thailand [19]. Despite the usefulness of the HIP-F in identifying areas of physical health risk, it is unclear if this resulted in any meaningful clinical benefits for study participants. An instrument has clinical utility when recommendations that are made based on the established risk factors are actually followed. However, it was beyond the scope of this study to ascertain the rate of follow-up actions. Therefore, a future study is now required to demonstrate that the HIP-F has utility beyond establishing risk factors/recommending actions, i.e., that it results in actual changes in patients’ behaviour, treatment and physical health state [72]. Therefore, in order to more conclusively demonstrate the clinical utility and efficacy of the HIP-F, future prospective studies should compare the outcomes of patients who have completed the HIP-F with those engaged in treatment as usual using a randomised controlled clinical trial.

### Strengths and limitations of the study

The study has methodological limitations that potentially limit the validity of the results and the generalisability of the findings. First, the HIP tool measures physical health across several items which may not correlate with each other [73]. Second, the procedure of cognitive debriefing and pilot testing based on Sousa’s and Rojjanasrirat’s [33] recommendations could not be carried out in full. Due to the small sample size and type of the instrument, our cognitive debriefing procedure was not piloted prior to the full pilot test and not all statistical methods to evaluate psychometric and reliability of instruments were appropriate (e.g. correlation coefficients, scale/tem analysis, calculation of sensitivity) [24, 33]. Third, some results of this study may be subject to reporting and social desirability bias because health behaviour is a self-reported measure, and there may be a tendency for participants to present an unrealistically positive picture of their health behaviours to the nurse working with them [74]. Fourth, convenience sampling was used in this study to recruit the participants, which is likely to introduce selection bias toward persons who are more motivated to discuss health issues [25, 75]. Similarly, selection bias can also be relevant for nursing staff because we are not fully aware how the nurses decided which patients they would invite to participate in the study. Fifth, despite all participants in the pilot testing had a psychotic disorder diagnosis categorised with ICD-10 (F20–29), the sample size was quite minor which might limit the generalisation of the results to all patients with severe mental illness. Sixth, the pilot testing data were collected by 17 individual nurses, which may cause variation in the results. For example, it is unclear if waist circumference was measured at the right point for all participants. Finally, some concepts used in the tool, such as BMI (body mass index), may not be frequently used in some Finnish hospital services, which may have negatively affected the clinical utility of these items.

Despite these limitations, the study has some strengths, specifically that the sample sizes for the content validity expert panel (*n* = 9) and in the pilot testing (*n* = 47) were adequate, and the heterogeneous sample seems to broadly represent the Finnish patient group relatively well. For example, the mean age of the participants is in line with the mean age of patients treated in psychiatric hospitals in Finland [76]. The correlative validity of the tool also seems to be desirable since the results are similar to previous HIP studies [19–22]. The response rate (55%) in the pilot testing was moderate-to-good, and the whole research process was carefully followed. Therefore, the overall results of the study can be considered as relatively dependable.

## Conclusions

The results demonstrate that the Finnish HIP has moderate content validity and preliminary clinical utility for evaluating the physical health and health behaviours of people with SMI in Finland. The findings provide additional evidence that the health behaviours and physical health state of people with SMI in Finland are major clinical problems that require urgent attention. The study findings also provide further support that people with SMI will engage effectively in comprehensive health checks. However, there seems to be a need for training among staff members in order to reinforce the rationale to focus on some of the less obvious areas of physical health risk and to support the effective use of the HIP-F in clinical practice.

## Abbreviations

15D: A 15-dimensional measure of health-related quality of life (HRQOL)
BMI: Body mass index
BPM: Beats per minute
cm: Centimetre
CVI: Content validity index
df: Degrees of freedom
EQ-5D: Health status measure by the EuroQol Group
F: Female
GAF: Global Assessment of Functioning
HDL: High-density cholesterol
HIP: Health improvement profile
HIP-F: Finnish health improvement profile
HUS: Helsinki University Hospital
IBM SPSS: IBM Statistical Package for Social Sciences
I-CVI: Item content validity index
IQR: Interquartile range
LDL: Low-density cholesterol
M: Male
Md: Median
Mmol/L: Millimole per litre
PHC: The physical health check
SAS-SMI: Social adjustment scale for the severely mentally ill
S-CVI: Scale level content validity index
S-CVI/Ave: Scale level content validity index/average
SD: Standard deviation
SF-12: The 12-item short form health survey
SMI: Severe mental illness
TC: Total cholesterol
TG: Triglycerides
WHOQOL: The world health organization quality of life
